# A Randomized Clinical Trial of Elemental Zinc Add-on Therapy on Clinical Outcomes of Patients with Chronic Rhinosinusitis with Nasal Polyposis (CRSwNP)

**DOI:** 10.22037/ijpr.2019.1100767

**Published:** 2019

**Authors:** Nader Akbari Dilmaghani, Nadereh Alani, Sepideh Fazeli

**Affiliations:** a *Department of Otolaryngology, Head and Neck Surgery, Loghman Hakim Educational Hospital, School of Medicine, Shahid Beheshti University of Medical Sciences, Tehran, Iran.*; b *Hearing Disorders Research Center, Shahid Beheshti University of Medical Sciences, Tehran, Iran.*

**Keywords:** Chronic rhinosinusitis, Nasal polyposis, Zinc, Lund-Kennedy score, Lund-Mackay score

## Abstract

Recent studies suggest a relationship between zinc deficiency and inflammation. In the present study, we studied the effect of oral zinc supplementation on clinical improvement of chronic rhinosinusitis with nasal polyposis. In this single-blind randomized controlled trial, 44 patients with chronic rhinosinusitis with polyposis referring to ENT clinic of the Loghman Hakim hospital during 2013-2014 were randomly allocated in two groups. The treatment group (n = 28) was treated with a four-drug fixed-dose regimen (FD_FDR) consisting of oral dexamethasone (0.02 mg/kg), fluticasone nasal spray, fexophenadine 60 mg daily, montelukast 10 mg daily plus 220mg zinc sulfate capsules containing 55 mg elemental zinc, b.d., and the control group (n = 16) received the FD_FDR without supplemental zinc, for six weeks. After sixth week, two groups were compared regarding clinical outcomes based on theSNOT20 (Sinonasal outcome test) questionnaire, the general health questionnaire (SF12), the Lund-Mackay, and the Lund-Kennedy scoring systems. In the treatment group, serum zinc levels were significantly increased compared to those at the baseline (1.33 fold-increase; *p *= 0.0002). Within groups analysis revealed a significant reduction (*p *< 0.01) in LM and LK in both treatment (55% LM; 50% LK) and control groups (45% LM; 53% LK). Incontrast, between groups analysis revealed no significant differences in the LM and LK. The treatment group showed a mild superiority in general health improvement compared to that of the control group. Add-on therapy with supplemental zinc sulfate was not associated with significant improvement in patients with chronic rhinosinusitis with nasal polyposis (CRSwNP). The advantage of zinc supplementation on the general health improvement of the patients with CRSwNP requires further assessments.

## Introduction

Chronic rhinosinusitis (CRS) is characterized by inflammatory reaction of the membrane of paranasal mucous, particularly by proliferation of inflammatory cells in the lamina propria which causes edema, fibrosis, or epithelial degradation (1, 2). In spite of initiating appropriate treatment, the inflammation lasts for at least four weeks, without an acute episode (3). The symptoms include nasal irritation, rhinorrhea or post nasal discharge, nasal congestion, and blockage and/or facial pain (4, 5). Furthermore, mucosal thickening, polypoid changes, and remarkable eosinophilia occur (3). In case of sever inflammation, nasal polyps infiltrated by inflammatory cells, mainly eosinophils, complicate the issue. Moreover, it forms pseudocysts consisting plasma exudation and albumin retention in the lamina propria (6). Microbial colonization is also found in sinusitis with intense eosinophil-dominated inflammation, nevertheless it remains unclear whether bacterial colonization is the first step prior to inflammation or it is resulted from outflow obstruction caused by polyps (7). Chronic rhinosinusitis (CRS) is one of the most common health care problems responsible for a large number of office visits, which adversely affects the quality of life (8). The exact etiology of CRS is not clear, therefore, there is more difficultly in treatment of the patients with CRS, using a divers class of drugs such as antibiotics, anti-inflammatory drugs, and systemic or topical corticosteroids but the efficacy is controversial (8, 9). Consequently, seeking appropriate treatment to manage the patients with CRS seems to be necessary. A possible option as a treatment supplement could be zinc. Although, there are insufficient evidences to recommend the use of zinc preparations in the patients with CRS, recent researches indicate that zinc supplementation can decrease incidence of many type of infections, vascular endothelial cell activation, oxidative stress, and nuclear factor-kappa B (NF-kB)-DNA binding in mononuclear cells (10-14). Furthermore, it improves T-helper cell function. According to a study in Turkey the serum levels of zinc were lower in the patients with CRS in comparison with the normal population (15). The present study was designed to investigate the impact of zinc supplement as add-on therapy of the patients with CRS especially those with polyposis (CRSwNP). 

## Experimental

In this single-blind randomized, controlled study, 56 patients with CRSwNP based on the 2012 European position paper on rhinosinusitis and nasal polyps (EPOS 2012) definitions for inclusion criteria for adult, referred to the ear, nose, and throat (ENT) clinic of Loghman Hakim Hospital during 2013 were randomly assigned to the treatment and control groups (16). The study procedure was explained for all patients and an informed written consent was obtained. Moreover, the ethics committee of the Shahid Beheshti University of Medical Sciences approved the study protocol (SBMU.REC.1392.270).

**Table 1:** Lund-Mackay scoring system for sinus CT scan

**Paranasal sinus right sinuses left sinuses**
frontal sinus
anterior ethmoidal cells
posterior ethmoidal cells
maxillary sinus
sphenoid sinus
ostiomeatal complex
Total
0 (no abnormality), 1 (partial opacification) or. 2 (complete opacification)

**Table 2:** Lund-Kennedy scoring system in nasal endoscopy

**Feature right nasal cavity left nasal cavity**
Polyp (0, 1, 2)
Edema (0, 1, 2)
Discharge (0, 1, 2)
Total

**Table 3:** Demographic, clinical, and laboratory characteristics of patients in Treatment and control groups

**Characteristics**		**RoutineTx(n = 16)**	**RoutineTx+Zinc(n = 28)**	***p*** **-value (between groups)**
Age		42.25 12.49	38.21 16.13	0.393
Sex	Male	10 (62.5%)	8 (28.6%)	0.028
	Female	6 (37.5%)	20 (71.4%)	
Zinc	Before	93.5 (86-113)	89 (87-96)	0.377
	After	108 (78-147)	118 (104-149)	0.088
	*p*-value (within groups)	0.637	0.0002	
Lund Kennedy endoscopic scoring	Before	8.5 (5-12)	8 (4-8)	0.296
	After	4 (2-8)	4 (2-7)	0.591
	*p*-value (within groups)	0.004	0.002	
	Non-Inferiority Power	100%		
Lund MackayCT scoring	Before	22.5 (17.5-24)	20 (11-23)	0.093
	After	12.5 (11-13)	9 (4-23)	0.231
	*p*-value (within groups)	0.004	0.0002	
	Non-Inferiority Power	100%		
SNOT20	Before	56.0 (38-71.5)	54 (42-64)	0.845
	After	34.5 (27.5-46.5)	33 (24-38)	0.144
	*p*-value (within groups)	0.013	0.00002	
	Non-Inferiority Power	100%		
General health	Before	26.5 (23.5-32)	26 (23-29)	0.555
	After	23.5 (20.5-24)	24 (23-25)	0.188
	*p*-value (within groups)	0.108	0.026	
	Non-Inferiority Power	100%		

**Figure 1:**
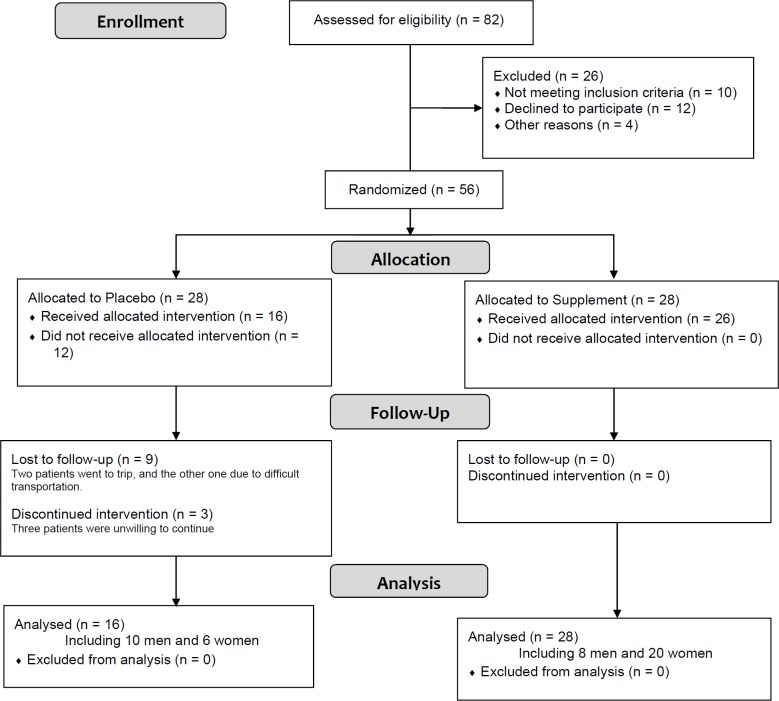
Flowchart of the trial

**Figure 2:**
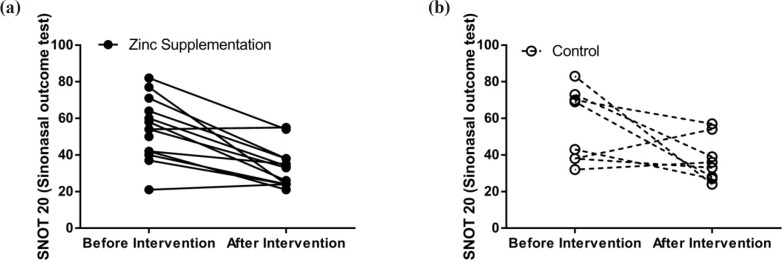
Comparison of SNOT20 before and after treatment in both groups

**Figure 3:**
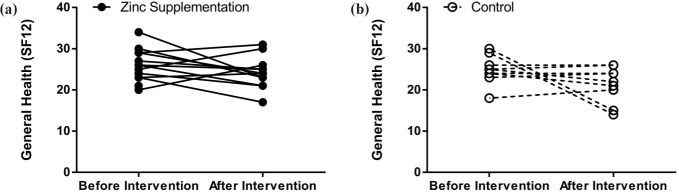
Comparison of SF12 before and after treatment in both groups

The trial was submitted in Iranian Registry of Clinical Trial at www.irct.ir and received (IRCT code: IRCT2016092018143N3).

The demographic data including sex, age, and subjective clinical status of patients were obtained based on SNOT20 questionnaire. SNOT20 is a patient-reported measure of outcome in sino-nasal disorders such as rhinosinusitis and nasal polyposis and includes items related to four domains: psychological function, sleep function, rhinological symptoms, and ear and/or facial symptoms (17). The findings of the paranasal CT scan were scored according to Lund-Mackay (LM) scoring system (Table 1) (18). Moreover, the sinonasal endoscopy results were accomplished and were scored based on Lund-Kennedy (LK) scoring system (Table 2) (19).

The first group of the patients (n = 28) received oral dexamethasone for three weeks (with initial dose of 0.02 mg/kg and tapered in 3 weeks), fluticasone 50 mcg spray/2puff/daily, fexophenadine tablet 60 mg daily, montelukast 10 mg daily, plus daily intake of two 220 mg zinc sulfate capsules equivalent to 55 mg elemental zinc, along with nasal irrigation with normal saline 2 times/day. The second group of the patients (control group, n = 28) received the same treatment regimen without zinc sulfate for six weeks (16, 20). The study staff (site investigators and trial coordinating center staff) was unaware of the treatment allocation. At the start of the treatment and at the end of six weeks both groups were compared regarding clinical state based on SNOT20 questionnaire, general health according to patient health questionnaire (SF12) and Lund-Kennedy as well as Lund-Mackay scores. Unfortunately, within the control group 12 (43%) of the patients did not complete the study and were excluded.

All the patients were instructed not to take any supplemental zinc product and also not to change their usual diet during the study period. To confirm medication adherence of the patients, plasma zinc levels were measured at baseline and at the end of the study. 

*Statistical analysis*

Categorical data are presented descriptively as frequency and percentages. Quantitative data with normal distribution (based on Shapiro-Wilk test) are expressed as mean ± SD, and non-normally distributed variables are shown as median and interquartile range (IQR). Data were analyzed by the Statistical Package for the Social Sciences (SPSS), version 20, IBM™. For between-group comparisons the independent samples *t*-test and the Mann-Whitney U test were used. The paired *t*-test and the Wilcoxon test were used for within-group comparisons. Furthermore, the Chi-square or the Fisher’s exact tests were employed to compare categorical variables between the study groups. The level of statistical significance was determined at *p *< 0.05. Moreover, the observed power of non-inferiority tests of comparing mean changes of treatment *vs. *the control groups were determined by NCSS PASS (Power Analysis and Sample Size), version 11 (21, 22).

## Results

In this RCT, 44 patients including 16 patients in routine treatment group ( 10 men and 6 women) and 28 patients in routine plus zinc treatment group (6 men and 20 women) were compared (mean age 39.68 ± 15.06). Figure 1 shows the flowchart of the trial. Difference between treatment and control groups regarding sex and age distribution were not statistically significant (Table 3).There were also no significant difference between baseline Lund scores (endoscopic and CT) of two groups (Table 3). However, the Lund scores decreased significantly after the treatment in the both treatments (55% LM, 50% LK; *p *< 0.01) and control groups (45% LM, 53% LK; *p *= 0.004). In the treatment group, serum zinc level significantly increased from median (IQR) 89 (87-96) mcg/dL to 118 (104-149) mcg/dL (*p *= 0.0002), while it did not dramatically change in the control group (Table 3).

Differences between baseline clinical status, determined by the SNOT20 questionnaire, and baseline general health (SF12), based on the patient health questionnaire, of the two study groups were not significantly regarded. SNOT20 scores were significantly higher in the both treatment (38.89%, *p *= 0.00002) and control (38.39%, *p* = 0.013) groups compared to those of the baseline. In the control group, general health status did not change during the study period; however, in the treatment group it showed improvement (Figures 2 and 3).

## Discussion

The treatment of CRS is more challenging for both physicians and patients. Traditionally, it is medically treated using systemic antibiotics, topical and systemic corticosteroids, antileukotriene agents, mucolytics, antihistamines, and saline nasal irrigation (23-25). However, previous studies have provided controversial results, and did not introduce an effective drug for CRS treatment (23-25). Therefore, the clinicians have already considered new agents for CRS treatment. In this study, for the first time, we evaluated the possible effect of zinc supplement on CRS. In both of the control and treatment groups, the mean of Lund score (endoscopic and CT) decreased significantly after a six-week treatment. In addition, general health of the patients improved in the Zinc group. Also, after six weeks of treatment, clinical status of the patients in the control and treatment groups improved significantly. Although, the impact of zinc on CRS has not yet been assessed, Unal *et al. *revealed that the patients with CRS had lower serum zinc levels (15). Another study by Gulani *et al*. detected decreased serum zinc levels in children with otitis media (26). Similarly, other studies showed zinc increases the immunity system function and decreases the incidence of infective diseases such as diarrhea and other GI infections (27-29). In accordance with these findings, Prasad *et al. *observed that zinc increases the function of both cellular and humoral immune systems (13). In conclusion, we revealed that addition of zinc supplement was not much effective on treatment of patients with CRSwNP, although, general health status of patients was improved with zinc supplementation.

As a limitation, we could not follow all of the 28 patients in the control group through the 6 weeks study period. This should be acknowledged in the interpretation of our findings. Nevertheless, the high non-inferiority power of this study to find similar effectiveness of zinc supplementation and current therapies for radiologic/endoscopic improvements of CRSwNP patients, in addition to mild superiority of zinc supplementation on general health improvement of the patients is encouraging. Further longitudinal investigations with more powerful designs are recommended.
